# Rhizosphere Bacterial Community Response to Continuous Cropping of Tibetan Barley

**DOI:** 10.3389/fmicb.2020.551444

**Published:** 2020-11-30

**Authors:** Youhua Yao, Xiaohua Yao, Likun An, Yixiong Bai, Deqing Xie, Kunlun Wu

**Affiliations:** ^1^Academy of Agricultural and Forestry Sciences, Qinghai University, Xining, China; ^2^Qinghai Key Laboratory of Hulless Barley Genetics and Breeding, Xining, China; ^3^Qinghai Subcenter of National Hulless Barley Improvement, Xining, China

**Keywords:** Tibetan barley, continuous cropping, rhizosphere soil, bacterial community structure, predictive functional profiling

## Abstract

Long-term continuous cropping influences the nutrient of soil and microbiome of the rhizosphere, resulting in the yield decrease of crops. Tibetan barley is a dominant cereal crop cultivated at high altitudes in Tibet. Its growth and yield are negatively affected by continuous cropping; however, the response of the rhizosphere microbial community to continuous cropping remains poorly understood. To address this question, we investigated the bacterial community structure and conducted predictive functional profiling on rhizosphere soil from Tibetan barley monocropped for 2–6 years. The results revealed that long-term continuous cropping markedly decreased total nitrogen and available nitrogen in rhizosphere soil. Illumina high-throughput sequencing of 16S rRNA genes indicated that the bacterial community was altered by continuous cropping; operational taxonomic units (OTUs), Shannon index, and Faith Phylogenetic Diversity decreased with increasing monocropping duration. Relative abundances of family *Pseudomonadaceae*, *Cytophagaceae*, and *Nocardioidaceae* were significantly increased, while those of *Chitinophagaceae* and *Sphingomonadaceae* were significantly decreased (all *p* < 0.05). Besides, continuous cropping significantly increased the abundance of bacteria associated with chemoheterotrophy, aromatic compound degradation, and nitrate reduction (*p* < 0.05). Generalized boosted regression model analysis indicated that total nitrogen was the most important contributor to the bacterial community diversity, indicating their roles in shaping the rhizosphere bacterial community during continuous cropping. Overall, continuous cropping had a significant impact on the structure of bacterial communities in rhizosphere soil of Tibetan barley, and these results will improve our understanding of soil bacterial community regulation and soil health maintenance in Tibetan barley farm systems.

## Introduction

High altitude ecosystems are generally characterized by low temperature, variable rainfall, reduced atmospheric pressure, and soil nutritional stress. Cold in high altitude region is the major factor that has confounded effects on both microbial biodiversity and soil physicochemical properties (Kumar et al., 2019). Plants and microbes are co-evolved and interact with each other in the environment. Thus, high altitude regions are the research center in understanding the interactions between certain microbes and the plants cultivated in the cold environment (Rawat et al., 2020). Lower species richness and diversity were observed in soil with higher elevations (Bryant et al., 2008; Wang et al., 2015). In a study of three vertical climate zones on the Qinghai-Tibetan Plateau, China (av. 4,000 m above sea level), soil bacteria exhibited more apparent elevational zonation features and decreased diversity patterns with increasing elevation (Wang et al., 2015). However, the microbial community structure of rhizosphere soil in high altitude region is less understood (Praeg et al., 2019).

Continuous cropping, defined as a single crop being cultivated in the same field year after year, is commonly practiced in China due to limited arable lands and lower associated agronomic management costs compared to rotation cropping (Zhang and He, 2004; Chen et al., 2009). However, long-term monoculture cropping can lead to serious soil sickness. Previous studies reported that continuous cropping often leads to restricted growth and crop yields with different time-scales (Ventura and Watanabe, 1978; Lua et al., 2017; Zhao et al., 2017). Furthermore, Changes in the diversity and composition of soil microorganisms by continuous cropping can disrupt ecosystem function, balance, and health; in turn, such phenomena negatively affect soil productivity and ultimately lead to reduced plant biomass and yield (Chen et al., 2012). Dynamic successions of the rhizosphere microbial community during continuous cropping have been identified in soybeans (Bai et al., 2015), cotton (Wei and Yu, 2018), peanut (Chen et al., 2014), and cucumber (Ali et al., 2019). However, these crops are usually cultivated in normal altitude regions, and the role of continuous cropping in shaping the microbiome structure of plants cultivated at high altitudes remains poorly investigated. Moreover, previous studies have proven that continuous cropping disturbed the microbial community structure in the rhizosphere soil, and loss of biodiversity has been identified as a common phenomenon in soil with long-term continuous cropping (Li et al., 2010, 2018; Chen et al., 2014). But, the effects of continuous cropping on the functional groups of rhizosphere microbe are less explored.

Tibetan hulless barley (*Hordeum vulgare* L., qingke) has been completely adapted to the extreme plateau climate after thousands of years of domestication and cultivation on the Qinghai-Tibetan Plateau, China (av. 4,000 m above sea level) (Zeng et al., 2018). Tibetan barley accounts for approximately 70% of the farming land in Tibet and serves as a food staple, animal fodder, and ingredient in various foods (Choo, 2002). However, the sustainability and development of Tibetan barley production are impeded by conventional farming practices, such as continuous cropping (Zhang et al., 2012). We hypothesized that the long-term continuous cropping of Tibetan barley alters the bacterial community structure and function in rhizosphere soil, resulting in specific responses of rhizosphere soil microbes to continuous cropping. Thus, understanding the influence of continuous cropping on the microbial community in the soil rhizosphere is pivotal for designing effective farm management practices to relieve soil depletion associated with Tibetan barley cultivation.

In this study, Tibetan barley fields cultivated for various durations were used to investigate the effect of continuous cropping on the rhizosphere bacterial community. Dynamic succession of the bacterial community structure in the rhizosphere was explored by 16S rRNA gene high-throughput sequencing. Changes in the potential ecological functions of the rhizosphere bacterial community during continuous cropping were also investigated. These results provide a comprehensive understanding of the rhizosphere microbial response to continuous Tibetan barley cropping and may help improve farming strategies by regulating the structure and function of rhizosphere soil flora to reduce the negative impacts of continuous cropping.

## Materials and Methods

### Field Description and Yield Investigation

The study field was located in Qinghai province, China (37°21′N, 101°44′E) at an altitude of 2,870 m. The Qinghai-Tibet plateau features a cold, warm, and humid climate, with an average annual sunshine duration of 2,575 h, average annual temperature of 1.3°C, annual rainfall of 530–560 mm, and annual evaporation of 1,128–1,343 mm (Zhang et al., 2000). The soil in the field is Kastanozems according to the FAO classification (FAO, 1990), with 4.5 g kg^–1^ total nitrogen, 1.0 g kg^–1^ total phosphorus, 22.0 g kg^–1^ total potassium, 60.5 g kg^–1^ organic matter, 245 mg kg^–1^ available nitrogen, 20 mg kg^–1^ available phosphorus, and 200 mg kg^–1^ available potassium. The experimental field contained four plots, each 10.0 × 10.0 m in size. Starting in June 2011, Tibetan barley (*Hordeum vulgare* L., qingke) is an annual crop. In the period of 2011–2016, the plants were cultivated in April and harvested in August, yearly. Blended fertilizer with 22.5 g m^–2^ (NH_4_)_2_SO_4_ and 11.5 g m^–2^ urea was applied annually before seeding. About 60,000 seeds were sowed at each plot, and approximately 250 plant m^–2^ successfully matured. All other field management activities were performed according to local farming habits. Harvesting was performed annually when plants reached full maturity, and seeds were manually separated to measure yield.

### Rhizosphere Soil Collection and Physiochemical Analyses

Rhizosphere soil samples were collected annually during the flowering period. Approximately 15 plants sample were collected from five different sites using a “Z” pattern in each experimental plot. Then, the composite sample was constituted from the 15 plants. Rhizosphere soils were collected for analysis as reported (Li et al., 2018). Briefly, plants were carefully pulled from the ground and the conglutinating soil from the roots was mildly crushed and shaken to collect the soil located within the plant root surfaces. Quadruple soil and plant samples were collected from the four experimental fields. The rhizosphere soil was immediately transported on ice to the laboratory and stored at −20°C for DNA extraction and chemical analysis. Half of each combined soil sample was air-dried and filtered through a 0.149 mm sieve. The sample pH was determined using a pH meter with a glass electrode (FE20-Five Easy Plus^TM^, Mettler Toledo, Greifensee, Switzerland). Total nitrogen (TN), available nitrogen (AN), total phosphorus (TP), total potassium (TK), rapidly available phosphorus (RAP), and rapidly available potassium (RAK) were measured as previously described (Van Raij et al., 1986; Nichols and Wright, 2006).

### Illumina MiSeq Sequencing of Bacterial 16S rRNA Genes

Total genomic DNA was extracted from 0.25 g of rhizosphere soil using the MoBio Soil DNA isolation kit (MO BIO Laboratories Inc., Carlsbad, CA, United States) according to the manufacturer’s protocol. The bacterial V4–V5 region of the 16S rRNA gene was amplified using the universal prokaryotic primers 515F (5′-GTG CCA GCM GCC GCG GTA A-3′) and 907R (5′-CCG TCA ATT CMT TTR AGT TT-3′) (Zhou et al., 2011) with the addition of a 12 bp unique barcode and the required Illumina adapters. PCRs were performed in a 30-μL mixture containing 10 μL TaKaRa *Ex* Taq^®^ PCR premix (TaKaRa Bio Inc., Kusatsu, Japan), 0.5 μL forward primer (10 μM), 0.5 μL reverse primer (10 μM), 1 μL DNA template (20 ng), and 18 μL PCR-grade water under the following PCR conditions: initial denaturation at 95°C for 3 min, 35 cycles of denaturation at 95°C for 30 s, annealing at 54°C for 30 s, extension at 72°C for 1 min, and then a final extension at 72°C for 10 min. Samples were amplified in triplicate, and replicate PCRs for each sample were pooled and purified using a QIAquick Gel Extraction Kit (Qiagen Inc., Chatsworth, CA, United States). PCR products were then sequenced using an Illumina MiSeq system at Sangon Biotech Co., Ltd. (Shanghai, China).

### 16S rRNA Sequence Analysis

The raw paired-end reads were merged using FLASH Version 1.2.11 (Magoč and Salzberg, 2011). Merged sequences were subsequently analyzed using the bioinformatics pipeline of Quantitative Insights Into Microbial Ecology (QIIME) version 2018.6 (Caporaso et al., 2010). First, demultiplexing was summarized using demux^1^ to classify sequences from each sample. Then, primer sequences were removed using “cutadapt” in QIIME2. After that, chimeric sequences were removed and sequences were clustered using QIIME 2’s q2-feature-classifier plugin^2^ (Bokulich et al., 2018). The clean data were then clustered into OTUs at 97% similarity using UCLUST (Edgar, 2010), and the longest sequence within each OTU was selected as the representative sequence. Taxonomic assignment of each OTU was conducted using QIIME 2’s q2-feature-classifier plugin^3^ and the SILVA version 132 database (Quast et al., 2012). The OTU table was rarefied to the minimum sample count (36,836 sequences) for subsequent analysis, including alpha and beta diversities and species composition. Alpha-diversity was described using OTUs, the Shannon index, and Faith Phylogenetic Diversity (PD).

### Predictive Functional Profiling of Bacterial Communities Using 16S rRNA Data

The predicted ecological functions of the prokaryotic communities were analyzed by mapping the taxonomy into certain metabolic pathways using FaProTax software (Louca et al., 2016). The OTU table generated from 16S rRNA sequence analysis was matched to annotated OTU information with species information in the FaProTax database to determine the predicted ecological function.

### Statistical Analysis

ANOVA and Spearman’s correlations were analyzed using SPSS Statistical Software Package ver. 20.0 (SPSS Inc., Chicago, IL, United States). Correlations between the community composition and environmental parameters were also analyzed by RDA (redundancy analysis) using the “vegan” package in R software (version 3.5.1). Linear regression models in R software were used to analyze the dynamic tendency of geochemical parameters, taxonomic abundance, and functional profiles against years of continuous cropping. Pearson relationships between physicochemical properties of rhizosphere soils and predictive functional profiles of bacterial communities were explored by SPSS 20.0. A correlation between two items was considered statistically robust if the Spearman’s correlation coefficient (ρ) was >0.8 and the *P*-value was <0.05. Co-occurrence network visualization was conducted on the interactive platform of Gephi (Bastian et al., 2009). To accurately predict and explain the relationships between bacterial data and chemical variables, generalized boosted regression modeling (GBM) analysis (5,000 trees used for boosting, 10-fold cross-validation, and three-way interactions) was performed to quantitatively evaluate the relative influence of individual chemical factors on bacterial community diversities using the R package “gbm” function (De’ath, 2007).

## Results

### Tibetan Barley Yields and Physicochemical Properties of Rhizosphere Soils

Yields consistently decreased during 6 years of Tibetan barley monoculture, from 19.42 ± 0.74 kg ha^–1^ to 12.40 ± 0.45 kg ha^–1^ (Supplementary Figure S1). Environmental parameters of the rhizosphere soil were also influenced by continuous cropping, although rhizosphere soil pH exhibited no obvious changes throughout the study and was slightly alkaline (pH ranged from 8.06 to 8.91) (Table 1). The concentration of TN and AN decreased with continuous cropping. However, phosphorus (TP and RAP) and potassium (TK and RAK) fluctuated and did not exhibit increasing or decreasing trends throughout the experimental period (Table 1).

**TABLE 1:** Effects of continuous cropping on rhizosphere soil physicochemical characteristics.

	**Duration of continuous cropping (years)**				
**Physicochemical factors^*b*^**	**2**	**3**	**4**	**5**	**6**
pH	8.42 ± 0.38	8.45 ± 0.15	8.52 ± 0.31	8.36 ± 0.10	8.57 ± 0.22
TP (g⋅kg^–1^)	1.63 ± 0.10^*c*^	2.26 ± 0.13^*a*^	1.97 ± 0.11^*b*^	2.26 ± 0.07^*a*^	1.92 ± 0.05^*b*^
TK (g⋅kg^–1^)	20.14 ± 0.33^*c*^	22.85 ± 0.59^*a,b*^	22.01 ± 0.35^*b*^	23.26 ± 0.45^*a*^	22.95 ± 0.70^*a,b*^
AN (mg⋅kg^–1^)	194.25 ± 5.12^*a*^	189.25 ± 8.42^*a*^	118.75 ± 3.50^*b*^	130.00 ± 3.74^*b*^	88.75 ± 4.03^*c*^
RAP (mg⋅kg^–1^)	50.15 ± 1.92^*c*^	97.68 ± 3.31^*a*^	53.83 ± 1.30^*c*^	98.05 ± 2.95^*a*^	68.90 ± 6.74^*b*^
RAK (mg⋅kg^–1^)	74.18 ± 3.97^*b*^	80.33 ± 2.49^*a,b*^	79.78 ± 9.04^*a,b,c*^	87.78 ± 4.31^*a*^	61.43 ± 3.01^*c*^

*Values represent means ± standard deviation (*n* = 4). Different lowercase letters within the same column indicate significant differences among different continuous cropping durations at *P* < 0.05 according to Duncan’s test. TN, total nitrogen; TP, total phosphorus, TK, total potassium; AN, available nitrogen; RAP, rapidly available phosphorus; RAK, rapidly available potassium.*

### Dynamic Succession of the Rhizosphere Bacterial Community

**FIGURE 1:**
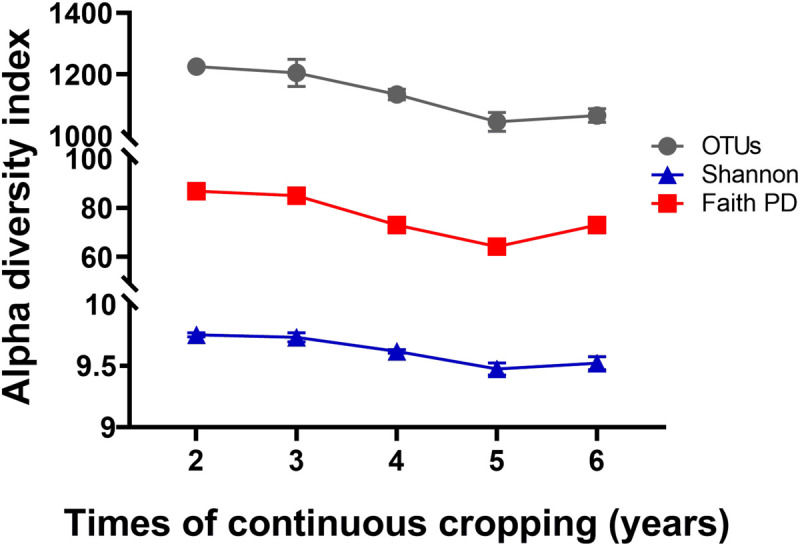
Effects of continuous cropping on bacterial alpha diversities for different continuous cropping durations. OTUs, Shannon index, and Faith PD in rhizosphere soils continuously cropped for 2 to 6 years; Symbols represent means ± SDs (*n* = 4).

**FIGURE 2:**
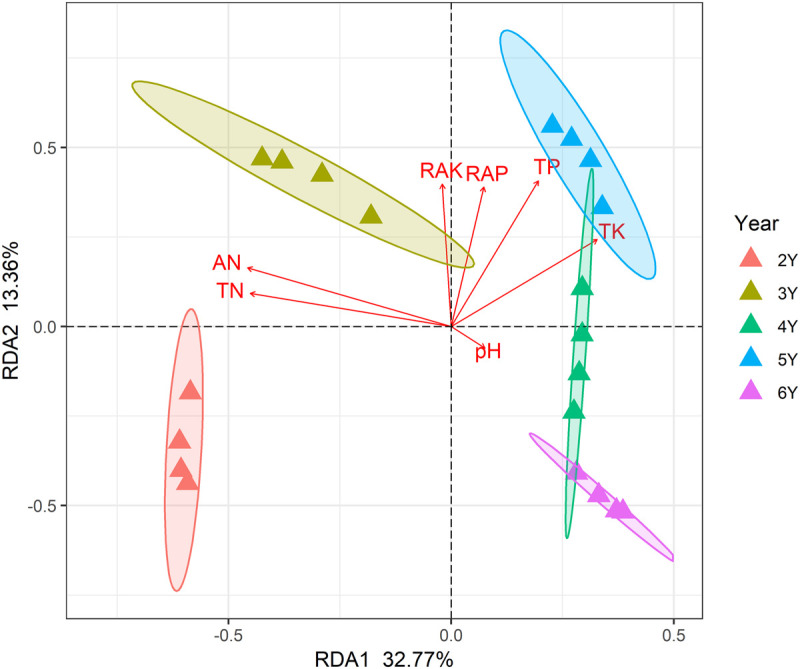
Redundancy analysis (RDA) biplot of bacterial 16S rRNA genes in the rhizosphere soils showing geochemical factors and samples.

A total of 799,745 qualified 16S rRNA gene sequences were obtained with an average of 58,670 sequences per sample. Each sample was rarified to 36,836 sequences to reduce the effect of sequence numbers on bacterial diversity and taxonomic composition analysis. The richness of the rhizosphere bacterial community declined during 6 years of continuous cropping (Figure 1). The number of OTUs decreased from 1,226 ± 7 at 2 years to 1,066 ± 22 at 6 years of continuous cropping, and the Faith PD decreased from 86.73 ± 0.71 to 73.04 ± 2.1. The Shannon index decreased from 9.75 ± 0.02 to 9.52 ± 0.05 with successive years of continuous cropping (*p* > 0.05, ANOVA) (Figure 1). RDA was performed to summarize the relationships between bacterial communities and soil variables. The results showed that the first two axes of the RDA accounted for 46.13% of the total community variance. The RDA plot showed a clear separation in the space among the samples at different years of continuous cropping. Bacterial communities from the second and third years (CC2Y and CC3Y) were separated from those from later years (CC4Y, CC5Y, and CC6Y) along the first RDA axis. This separation was influenced mainly by AN, TN, pH. The structure of CC2Y and CC3Y communities are related to lower pH and higher AN and TN values in contrast to CC4Y, CC5Y, and CC6Y communities. Communities from the last three years were separated along the second RDA axis, and especially those from the fourth and fifth years were related to higher TP and TK. CC3Y and CC5Y are related to higher RAK and RAP (Figure 2). By contrast, the impact of pH on bacterial community composition was minimal.

The predominant phyla in the rhizosphere soil were *Proteobacteria* (42.72% ± 1.69% relative abundance), *Bacteroidetes* (12.33% ± 1.95%), and *Actinobacteria* (11.2% ± 3.12%) (Supplementary Figure S2A). At the family level, the bacterial community was dominated by *Sphingomonadaceae* and *Xanthomonadaceae* (Supplementary Figure S2B). The relative abundance of *Sphingomonadaceae* fluctuated and tended to decrease from 9.47% ± 0.05% to 7.36% ± 0.30% throughout the experiment. The relative abundance of *Xanthomonadaceae* increased from 2.00% ± 0.09% to 5.64% ± 0.75% at 4 years of monocropping and was restored to 2.37% ± 0.14% after 6 years of continuous cropping (Supplementary Figure S2B). We also explore the changes of 20 most abundant OTUs during continuous cropping. The abundance of OTU1 (classified as Unclassified *Exiguobacterium*), OTU5 (sugarcane phytoplasma), OTU9 (Unclassified *Pseudonocardiaceae*), OTU11 (Unclassified *Xanthomonadaceae*), and OTU12 (Unclassified *Nocardioides*) were significantly increased after 6 years of continuous cropping. By contrast, the OTUs displayed significantly decreased, including OTU2 (*Sphingomonas jaspsi*), OTU14 (Unclassified *Brevundimonas*), OTU17 (Unclassified *Sphingomonadaceae*), and OTU20 (*Burkholderiales* bacterium X4) (Supplementary Table S1). Significant linear correlations between taxonomic abundance and cropping duration were only identified in the less abundant families (Supplementary Table S2). The relative abundances of *Pseudomonadaceae* (*r*^2^ = 0.844, *p* < 0.05, Supplementary Figure S3A), *Cytophagaceae* (*r*^2^ = 0.696, *p* < 0.05, Supplementary Figure S3B), and *Nocardioidaceae* (*r*^2^ = 0.656, *p* < 0.05, Supplementary Figure S3C) were positively related to the duration of continuous cropping. *Chitinophagaceae* (*r*^2^ = 0.695, *p* < 0.05, Supplementary Figure S3D) exhibited a significantly negative relationship with cropping duration. The dynamic succession of the above families suggested that functionalities carried out by affiliated bacteria were critical in the rhizosphere biotope during continuous cropping.

### Changes in Bacterial Community Functionality With Continuous Cropping Duration

**FIGURE 3:**
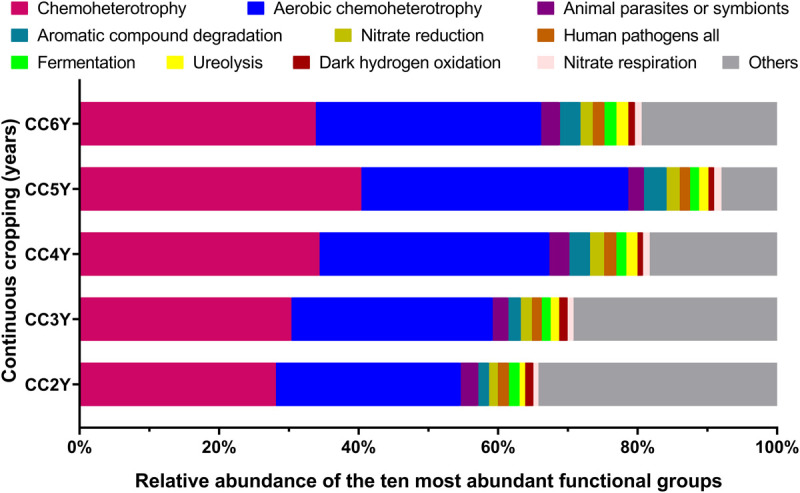
Distribution of bacterial ecological function in the FaProTax database for different continuous cropping durations. CC2Y, CC3Y, CC4Y, CC5Y, and CC6Y represent continuous cropping for 2, 3, 4, 5, and 6 years, respectively. Values represent means (*n* = 4). The 10 most abundant bacterial functional groups are shown.

**FIGURE 4:**
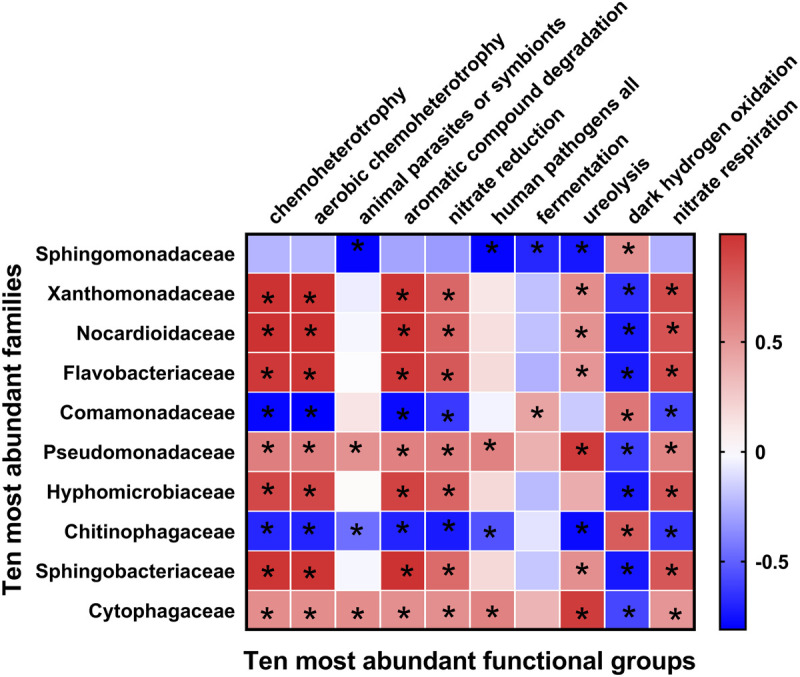
Heatmap of correlations between bacterial families with ecological functions in rhizosphere soil. Heatmap values ranged from +0.5 to −0.5. Values above/below zero represent positive/negative correlations between bacterial families and parameters analyzed. *P < 0.05 for the indicated comparisons.

Fifty-one ecological functions were annotated by FaProTax software, in which chemoheterotrophy (average abundance 33.42% ± 4.36%), aerobic chemoheterotrophy (31.79% ± 4.19%), animal parasites or symbionts (2.53% ± 0.28%), aromatic compound degradation (2.49% ± 0.73%), and nitrate reduction (1.69% ± 0.28%) were the predominant functional groups in the rhizosphere soil of Tibetan barley (Figure 3 and Supplementary Table S3). The results of linear correlation analysis revealed that the relative abundances of the taxa associated with chemoheterotrophy (*r*^2^ = 0.51), aerobic chemoheterotrophy (*r*^2^ = 0.53), aromatic compound degradation (*r*^2^ = 0.73), and nitrate reduction (*r*^2^ = 0.41) increased significantly with continuous cropping duration (all *p* < 0.05; Supplementary Figure S4, Table S4). Furthermore, significantly positive relationships were identified between abundant families and functions. However, dark hydrogen oxidation was only positively related to *Sphingomonadaceae*, *Comamonadaceae*, and *Chitinophagaceae* (all *p* < 0.05; Figure 4). Interestingly, the three families mentioned above were negatively related to the functional groups, which were positively correlated with other bacterial families, suggesting that the functionalities of *Sphingomonadaceae*, *Comamonadaceae*, and *Chitinophagaceae* were different from other bacterial families in the rhizosphere biotope of Tibetan barley.

### Linking Bacterial Community Structure and Functionality to Rhizosphere Soil Properties

**FIGURE 5:**
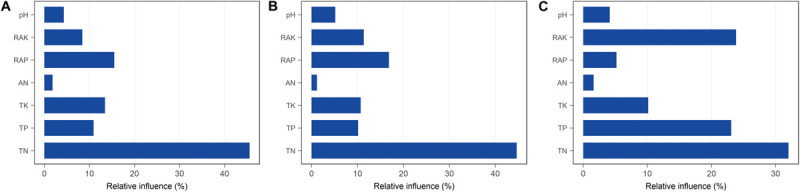
Relative influence (%) of individual physicochemical parameters on bacterial diversity in rhizosphere soil with different continuous cropping durations. **(A)** OTUs; **(B)** Shannon index; **(C)** Faith PD.

**FIGURE 6:**
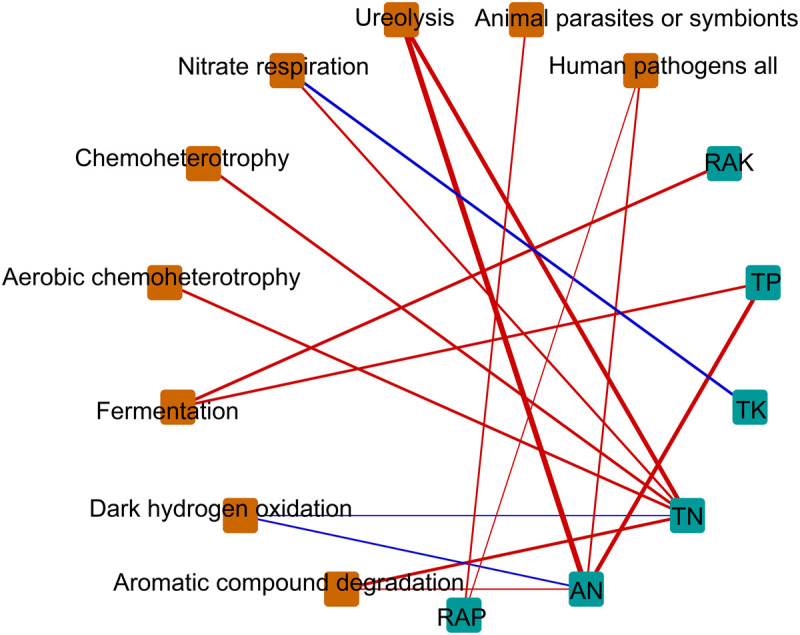
Correlation-based network analysis showing potential interactions between physicochemical parameters and ecological function. Node size is proportional to a functional group’s average relative abundance (log transformation) across all the samples. Lines connecting nodes (edges) represent positive (blue) or negative (red) co-occurrence relationships. TN, total nitrogen; TP, total phosphorus, TK, total potassium; AN, available nitrogen; RAP, rapidly available phosphorus; RAK, rapidly available potassium.

We hypothesized that bacterial communities in monocropped rhizosphere soil are shaped by innate environmental parameters. GBM analysis indicated that TN had the greatest relative influence on OTU numbers (45.55%), Shannon index (44.64%), and Faith PD (32.02%) of the bacterial communities studied (Figure 5), implying that nitrogen biotransformation was most influenced by changes in bacterial community diversity during continuous cropping. Pearson’s correlation coefficients between relative abundances of bacterial families and environmental parameters revealed that TN was positively correlated with the abundance of *Comamonadaceae* but negatively related to *Hyphomicrobiaceae* (all *p* < 0.01; Supplementary Figure S5). Furthermore, *Comamonadaceae* abundance was significantly negatively related to RAK (*p* < 0.01; Supplementary Figure S5). The correlation-based network analysis revealed that AN was positively related to dark hydrogen oxidation, and TK was positively related to nitrate respiration, but negative relationships were observed between other geochemical properties and the relative abundance of functional groups (all *p* < 0.05, Figure 6). Because pH remained constant throughout the experimental period, no remarkable relationship was observed between pH and ecological function. Some functional groups were significantly correlated with phosphorus and potassium concentrations in rhizosphere soil; for example, TK was significantly positively related to nitrate respiration, aromatic compound degradation, chemoheterotrophy, and aerobic chemoheterotrophy (Figure 6).

## Discussion

It has been suggested that continuous monoculture systems have negative impacts on soil function and sustainability (Liu et al., 2006; Chen et al., 2009, 2015; Nayyar et al., 2009; Bado et al., 2010; Li et al., 2010; Zhao et al., 2018). However, little is known about how soil geochemical properties are affected by continuous cropping in Tibetan barley. In this study, we found TN and AN were decreased with continuous Tibetan barley cropping (Table 1). Continuous cropping may alter soil structure and lead to the hardening of soil (Rezapour and Samadi, 2012) and hollowness of the tillable layer (Mikha and Rice, 2004), producing obstacles for nutrition transport from bulk soil to rhizosphere soil. The TN and AN tapering may be derived from the change of soil structure after long-term Tibetan barley continuous cropping. The ability of the rhizosphere soil to transform nitrogen elements decreases during continuous cropping, and the decreased TN and AN may attribute to the low yields of Tibetan barley in the fields. The grain yields of Tibetan barley ranged from 1.5 to 6.1 t ha^–2^ with different concentrations of urea N fertilization (Cui et al., 2016). According to the Standard of Qinghai Province (DB63/T1536-2020), the high, intermediate, or low yields of Tibetan barley are 5.25 to 6.00 t ha^–2^, 4.50 to 5.25 t ha^–2^, and 4.05 to 4.5 t ha^–2^, respectively. In this study, yields of Tibetan barley consistently decreased during 6 years of monoculture, from 4.37 ± 0.17 t ha^–2^ to 2.79 ± 0.10 t ha^–2^ (Supplementary Figure S1), and were all classified as low levels of crop productivity. In accordance with our results, previous studies reported that continuous cropping often leads to restricted growth and crop yields with different time-scales (Ventura and Watanabe, 1978; Lua et al., 2017; Zhao et al., 2018), which could result from the increase in autotoxicity of root exudates (Gao and Zhang, 1998), the accumulation of pathogens (Li et al., 2014), and the disturbance of the microbial community structure in the soil (Hamid et al., 2016). In our study, bacterial community structures and functions in rhizosphere soil were significantly altered by the continuous cropping of Tibetan barley, which may influence element transformation in the soil and nutrition uptake up by the plant.

Bacterial community diversity significantly decreased with the increased continuous cropping duration of Tibetan barley (Figure 1). Decreased microbial diversity was also observed in rhizosphere soil with continuous cropping of cucumbers, rice, ginseng, and peas (Reichardt et al., 1997; Yao et al., 2006; Nayyar et al., 2009; Tan et al., 2017). These findings indicated that diversity reduction in soil microbial communities with continuous cropping could be common. The results of the present study provided evidence that diversity, taxonomic richness, and evenness of rhizosphere soil bacteria were strongly influenced by continuous cropping (Figure 1). OTU numbers and Faith PD (phylogenetic diversity) for rhizosphere soil decreased significantly with an increased duration of continuous cropping. In addition, changes in soil chemical properties were likely to drive bacterial community changes in rhizosphere soil. We found that TN the most important factor explaining the alpha diversity reduction of the rhizosphere bacterial community during continuous cropping (Figure 5). Soil nitrogen availability is known to drive rhizosphere bacterial community assembly during plant growth (Bell et al., 2015), and available N, NO_3_^–^-N, and NH_4_^+^-N were the main environmental factors affecting the distribution of the community structure in intensive plant monoculture (Li G. et al., 2016).

Loss of biodiversity has been identified as a common phenomenon in soil with long-term continuous cropping. In this study, alpha diversities (OTU numbers and Faith PD indices) declined during 5 years of continuous cropping but increased at 6 years (Figure 1). Similar results have also been observed in soil with continuous cropping of cotton and tea (Li Y.C. et al., 2016; Wei and Yu, 2018; Xi et al., 2019; Liang et al., 2020). We inferred that changes in soil chemical properties could have contributed to the distinct variations in bacterial community structures, as soil chemical properties play critical roles in microbial community structures (Kuramae et al., 2012). It is also worth noting that changes in bacterial community diversity and structure in Tibetan barley monoculture might also be caused by root exudate effects. Root exudates directly influence the assemblage and activities of the rhizosphere microbiota. Plants not only provide nutrients for microbial communities but also contain a series of antimicrobial metabolites in their root exudates (Bais et al., 2006; Berg and Smalla, 2009). It has been confirmed that continuous cropping accumulates root exudates, which results in a microbial community structure shift (Reichardt et al., 1997; Strickland et al., 2015; Wu et al., 2019). Monocropping results in plant roots repeatedly releasing the same exudates in the same fields long term, which can result in significant colonization by certain pathogenic or beneficial microorganisms that utilize these substrates (Brimecombe et al., 2001). Thus, the continuous cropping system was speculated as a dominant factor disrupting the balance of the bacterial community structure in Tibetan barley rhizosphere soil.

Continuous cropping not only affects bacterial diversity but also bacterial community composition in rhizosphere soil. The abundances of *Pseudomonadaceae*, *Cytophagaceae*, and *Nocardioidaceae* increased significantly throughout the experiment (Supplementary Figure S3), indicating that the bacteria in these lineages were more adaptable to the continuous cropping environment. The relative abundance of *Pseudomonas* (in the family of *Pseudomonadaceae*) increased from 0 to 10-yr in soil with continuous cropping of tea (Li Y.C. et al., 2016). A previous study demonstrated that *Pseudomonas* density was closely related to plant growth (Rumberger et al., 2007). Furthermore, we found the relative abundance of *Pseudomonas* was positively related to most of the predicted functionalities of bacterial communities in rhizosphere soil (Figure 4). The above results indicated that bacteria affiliated with the family *Pseudomonadaceae* positively contributed to the effects of Tibetan barley continuous cropping. The relative abundance of *Actinobacteria* increased during continuous cropping, and *Xanthomonadaceae* increased at 4 years of monocropping and was restored after 6 years of continuous cropping (Supplementary Figure S3). Similarly, *Actinobacteria* was strongly enriched in the soil after long-term cotton continuous cropping (Xi et al., 2019). *Actinobacteria* take part in the global carbon cycle and break down soil organic matter (Upchurch et al., 2008). Thus, members of the phylum *Actinobacteria* in continuous Tibetan barley cropping soils may have a role in carbon transformation in rhizosphere soil. Moreover, high-throughput sequencing technology reveals that continuous cropping of *American ginseng* results in an increase of *Xanthomonadaceae* (Dong et al., 2017). However, in a study of consecutive monoculture of sweet potato, the abundance of *Xanthomonadaceae* in soil decreased considerably as the number of continuous cropping years increased (Li et al., 2018). A decrease in *Nocardioidaceae* abundance was observed in rhizosphere soil during continuous cropping of Tibetan barley (Supplementary Figure S3). The abundances of *Nocardioidaceae* decreased with the increasing years of consecutive monoculture in rhizosphere soils of *Achyranthes bidentata* (Wang et al., 2019). However, the functions carried out by *Xanthomonadaceae* and *Nocardioidaceae* in the soil are less understood, and their relationship with plant continuous cropping should be explored in future studies. *Chitinophagaceae* was the only family detected whose abundance significantly decreased with increasing continuous cropping duration (Supplementary Figure S3). However, the functions carried out by *Chitinophagaceae* warrant further exploration. Members of the genus *Sphingomonas* have raised increasing attention due to their ability for organic carbon degradation and their ubiquity in the environment (Bastiaens et al., 2000; Leys et al., 2004). OTUs classified as *Sphingomonas* (OTU2, OTU4, OTU6, and OTU8; Supplementary Table S1) dominated in the rhizosphere bacterial communities, suggesting their roles in utilizing organic carbon or root exudates in the rhizosphere soil of Tibetan barley during continuous cropping. It was also observed that phytoplasma (OTU5), exhibited an increasing pattern during continuous cropping. Phytoplasma is associated with the yellow leaf syndrome disease in sugarcane (Parmessur et al., 2002), however, the role of phytoplasma in the rhizosphere environment is less understood, and its response to continuous cropping should be illustrated in the future. In summary, we speculate that the functional capabilities of the microbiome can greatly affect the structural and functional diversity of the bacterial community during continuous cropping of Tibetan barley.

Overwhelming scientific evidence has demonstrated that the rhizosphere soil microbial community diversity and structure are strongly influenced by continuous cropping systems. However, the impact of continuous cropping on the functions of rhizosphere microbial communities of plants cultivated at high altitudes remains poorly understood. In this study, FaProTax software (Louca et al., 2016) was used to predict the ecological functions of the rhizosphere soil bacterial community during continuous cropping of Tibetan barley. The results indicated that chemoheterotrophy and aerobic chemoheterotrophy-related species were dominant in rhizosphere soil (Figure 3), which concurred with previous soil findings (Legrand et al., 2018; Che et al., 2019; Liang et al., 2020). We observed that the relative abundances of 16S rRNA gene sequences assigned to chemoheterotrophy and aerobic chemoheterotrophy increased significantly with continuous cropping duration (Supplementary Figure S4). In continuous cropping systems, root exudates, including the secretion of a diverse array of carbon-containing metabolites, such as sugars, amino acids, and phenolic acids, are easily decomposable and act mainly as carbon sources for soil microbes (Bais et al., 2006). Increasing evidence suggests that the continuous cropping of plants can enrich the soil in root exudates (Guo et al., 2011). Thus, the accumulation of root exudates may be a major reason for the increased chemoheterotrophs during continuous cropping of Tibetan barley.

Plant–soil feedback occurs in the duration of continuous cropping, with consequent effects on plants grown and soil microbial communities. Many secondary metabolites secreted by the plant can inhibit microbial growth by autotoxicity or allelopathy effects (Bennett and Klironomos, 2019). Thus, secondary metabolites should be another important factor in determining the bacterial community structure of rhizosphere soil under continuous cropping conditions. Long-term continuous cropping causes substantial enrichment of harmful secondary metabolites secreted by plants, which eventually leads to the changes in the structure and function of rhizosphere microbial communities (Shen et al., 2020). Long-term continuous cropping in soybean causes substantial changes to the types and amounts of secondary metabolites secreted by the roots, leading to an enrichment of harmful secondary metabolites (phenolic acids, benzene, and esters), which eventually results in autotoxicity (Cui et al., 2018). It was also showed that secondary metabolites (ferulic and *p*-hydroxybenzoic acids) influenced the microbial composition of the cucumber rhizosphere (Jin et al., 2020), and phenolic acids could change soil microbial composition over continuous cropping seasons in tobacco (Shen et al., 2020). Secondary metabolism is involved in the tolerance to drought and salinity in Tibetan barley (Ahmed et al., 2015; Yuan et al., 2018). However, the changes of secondary metabolites in the rhizosphere of Tibetan barley during continuous cropping have not been reported, and their effects on rhizosphere soil microbes should be explored in the future.

## Conclusion

In summary, our study demonstrated TN was the most important contributor to bacterial community diversity during continuous cropping, of which *Pseudomonadaceae*, *Cytophagaceae*, and *Nocardioidaceae* were key bacteria. Moreover, continuous cropping significantly increased the relative abundances of bacterial groups capable of chemoheterotrophy, aromatic compound degradation, and nitrate reduction. However, we only predicted bacterial function from a taxonomy assignment in this study. Further research should focus on obtaining direct evidence of changes in soil microbial functions in our continuous cropping system through approaches such as metagenomics or metatranscriptomic sequencing. The effects of continuous cropping on bacterial ecology in the rhizosphere of Tibetan barley should be given more attention to developing improved practices for continuous soil management and accelerated soil resource recycling in areas of high altitude.

## Data Availability Statement

The datasets presented in this study can be found in online repositories. The names of the repository/repositories and accession number(s) can be found below: https://www.ncbi.nlm.nih.gov/, PRJNA622628.

## Author Contributions

YY and XY carried out the molecular experiments, analyzed data, and wrote the manuscript. YY and LA carried out data analyses. YB and DX contributed to the field experiments and collected samples. KW conceived the study, contributed to the design, and interpreted the research. All authors read and approved the final manuscript.

## Conflict of Interest

The authors declare that the research was conducted in the absence of any commercial or financial relationships that could be construed as a potential conflict of interest.

## Footnotes

1 https://github.com/qiime2/q2-demux

2 https://github.com/qiime2/q2-dada2

3 https://github.com/qiime2/q2-feature-classifier

## Supplementary Material

Supplementary Figure 1 Effects of continuous cropping on Tibetan Barley yield. Values are means ± standard deviation (*n* = 4).

Supplementary Figure 2 Relative abundance of bacterial taxa in rhizosphere soil of Tibetan barley with different continuous cropping durations. **(A)** Relative abundances of the 10 most abundant bacterial phyla. **(B)** Relative abundances of the 10 most abundant bacterial families. Symbols represent means (*n* = 4).

Supplementary Figure 3 Relative abundances of *Pseudomonadaceae* **(A)**, *Cytophagaceae* **(B)**, *Nocardioidaceae* **(C)**, and *Chitinophagaceae* **(D)** plotted against years of continuous cropping of Tibetan barley.

Supplementary Figure 4 Relative abundance of ecological function plotted against years of continuous cropping of Tibetan barley. **(A)** chemoheterotrophy; **(B)** aerobic chemoheterotrophy; **(C)** aromatic compound degradation; **(D)** nitrate reduction.

Supplementary Figure 5 Spearman’s correlation heatmap based on population abundance and environmental variables for families with abundance >1%. AN, available nitrogen; TN, total nitrogen; RAK, rapidly available potassium; RAP, rapidly available phosphorus; TP, total phosphorus, TK, total potassium. Positive correlations (red), negative correlations (blue). **P* < 0.05 and ***P* < 0.01 for the indicated comparisons.

Supplementary Table 1 The changes of the 20 most abundant OTUs during continuous cropping.

Supplementary Table 2 Relative abundance of families against years of continuous cropping of Tibetan barley.

Supplementary Table 3 Dynamics of ecological function during continuous cropping of Tibetan barley.

Supplementary Table 4 Relative abundance of ecological function against years of continuous cropping of Tibetan barley.
