# Supplementary figures and images for: Rhizosphere Bacterial Community Response to Continuous Cropping of Tibetan Barley
Source: Front Microbiol. 2020 Nov 30;11:551444. doi: 10.3389/fmicb.2020.551444 (PMC7734106; doi:10.3389/fmicb.2020.551444)

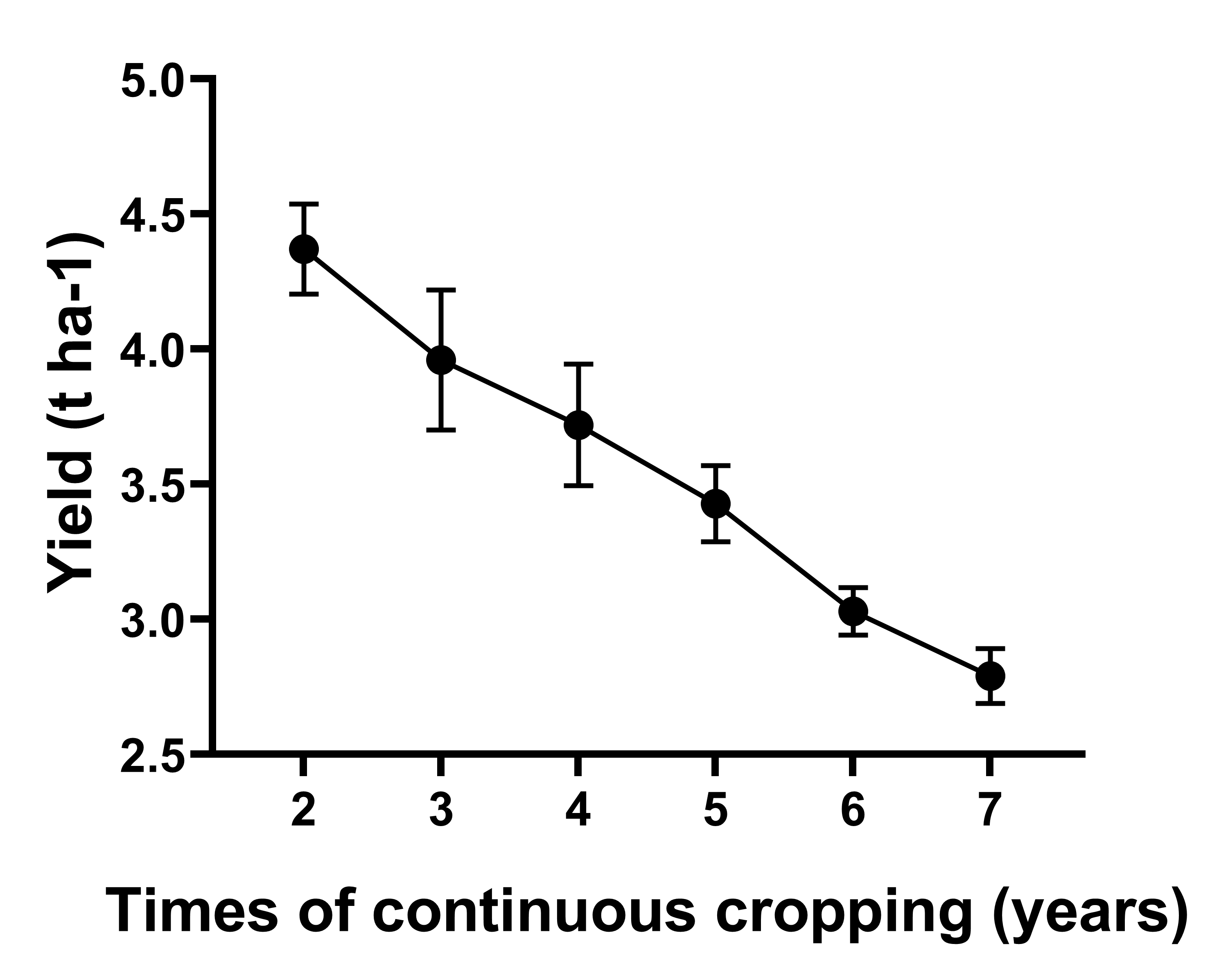

Supplement: Supplementary Figure 1 — Effects of continuous cropping on Tibetan Barley yield. Values are means ± standard deviation (n = 4). [file Image_1.TIF]

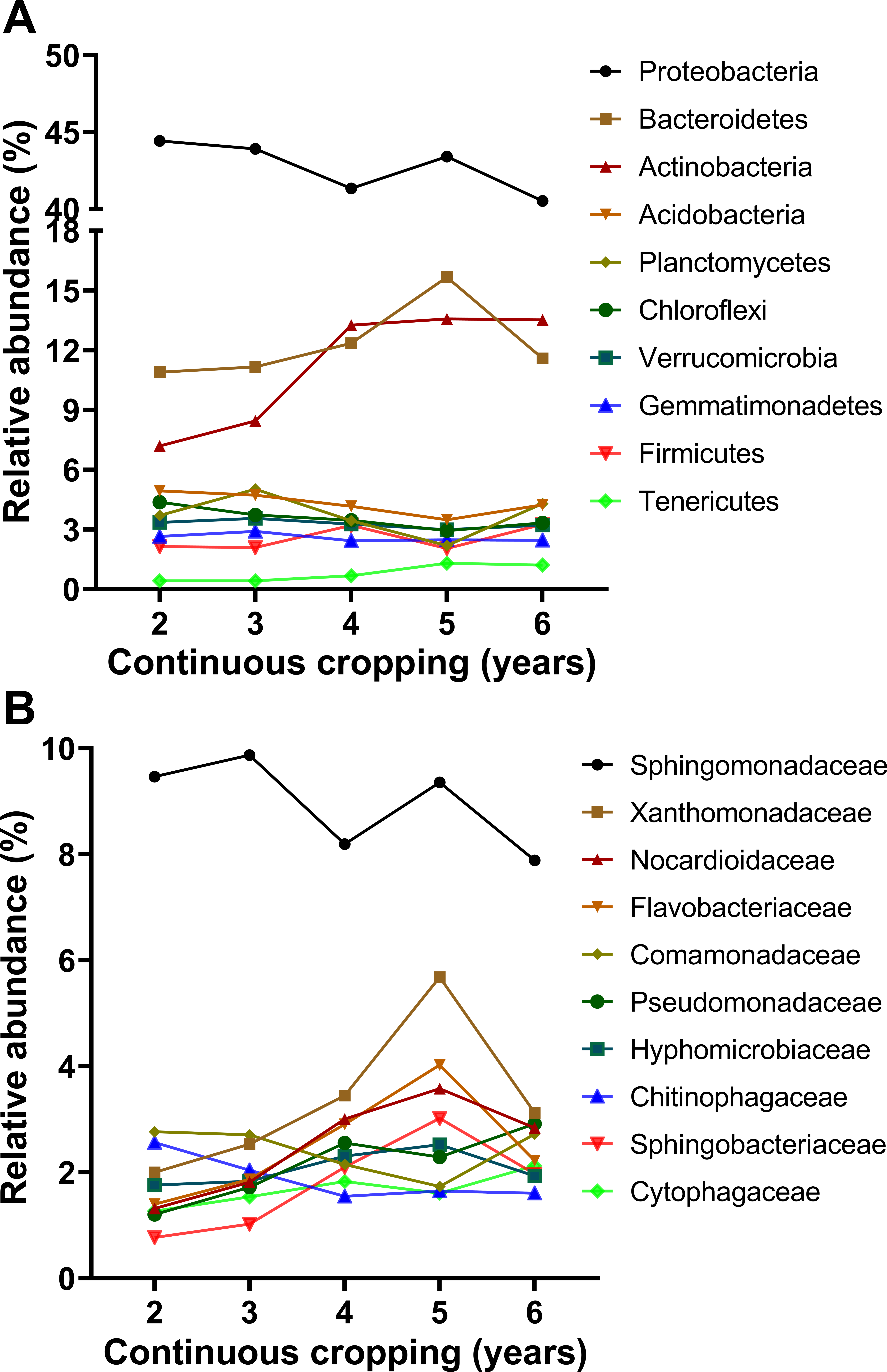

Supplement: Supplementary Figure 2 — Relative abundance of bacterial taxa in rhizosphere soil of Tibetan barley with different continuous cropping durations. (A) Relative abundances of the 10 most abundant bacterial phyla. (B) Relative abundances of the 10 most abundant bacterial families. Symbols represent means (n = 4). [file Image_2.TIF]

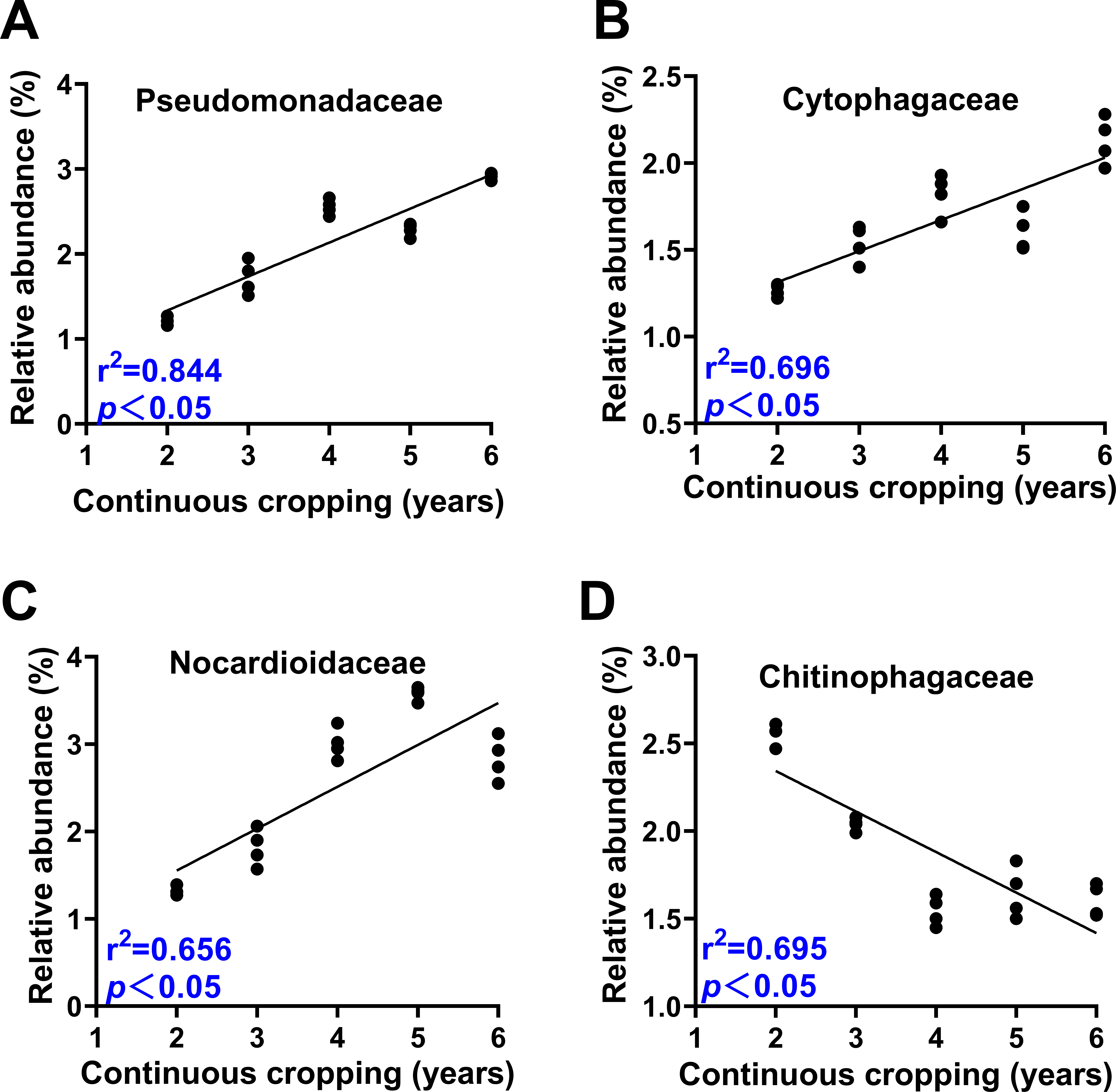

Supplement: Supplementary Figure 3 — Relative abundances of Pseudomonadaceae (A), Cytophagaceae (B), Nocardioidaceae (C), and Chitinophagaceae (D) plotted against years of continuous cropping of Tibetan barley. [file Image_3.TIF]

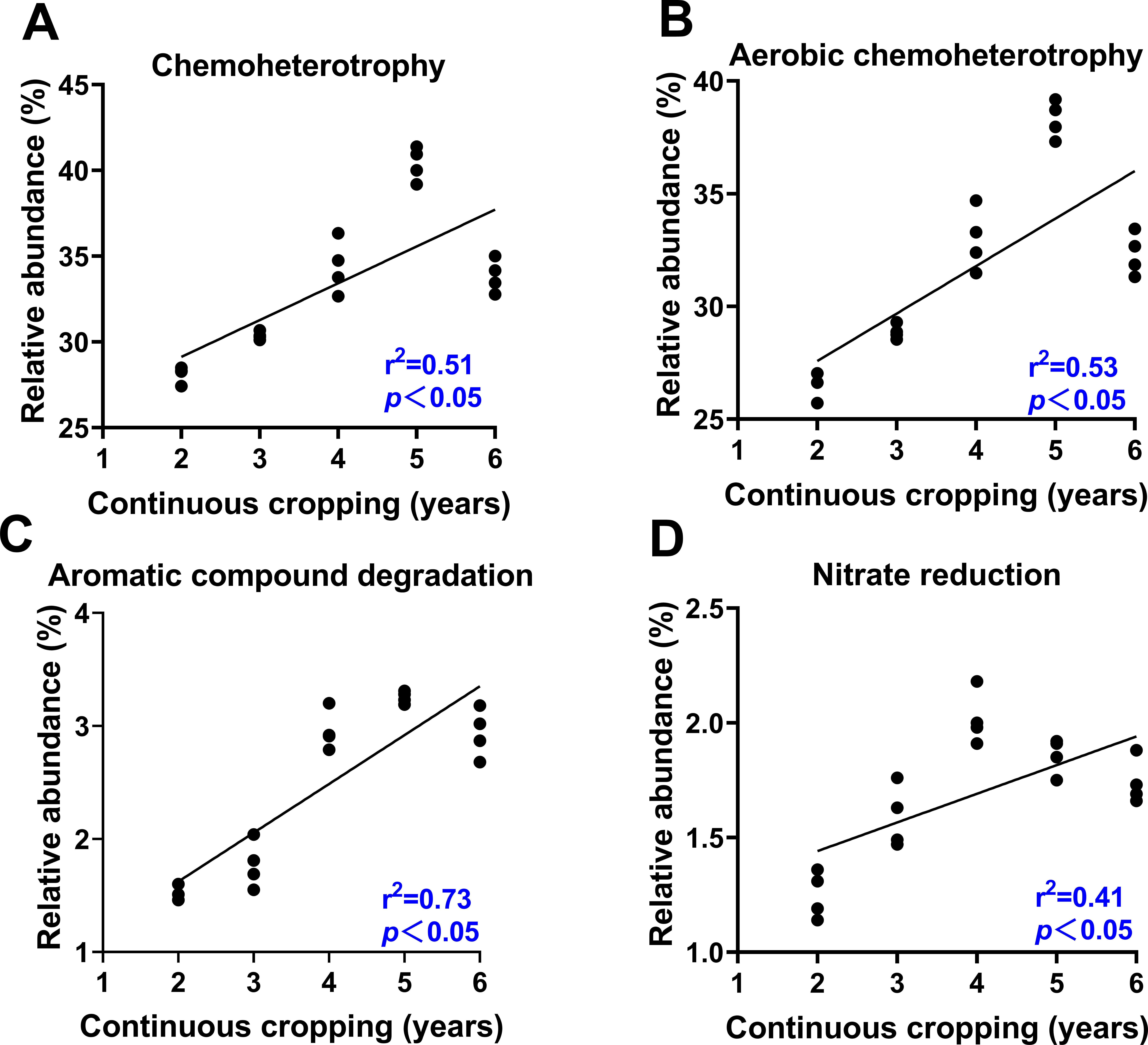

Supplement: Supplementary Figure 4 — Relative abundance of ecological function plotted against years of continuous cropping of Tibetan barley. (A) chemoheterotrophy; (B) aerobic chemoheterotrophy; (C) aromatic compound degradation; (D) nitrate reduction. [file Image_4.TIF]

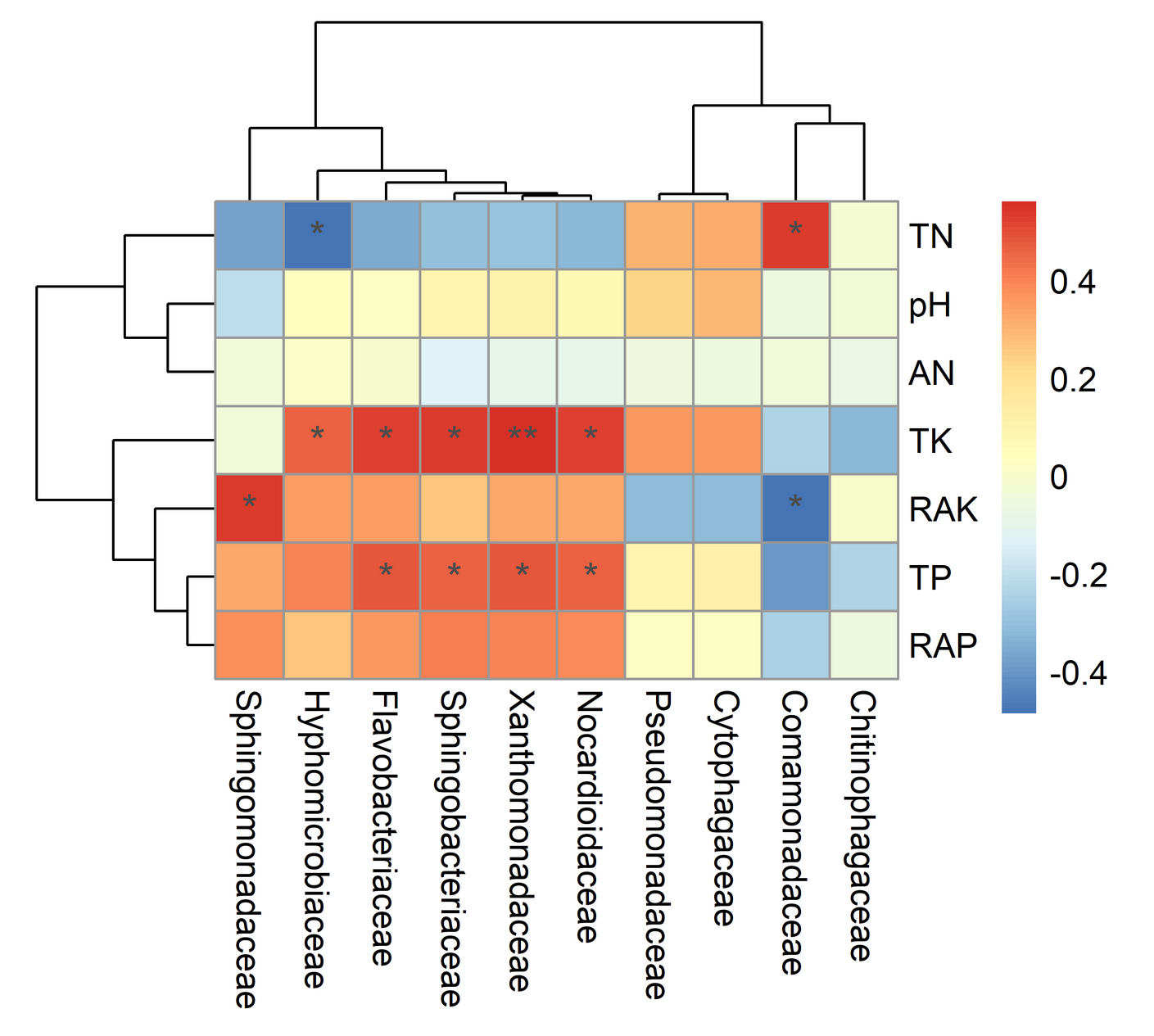

Supplement: Supplementary Figure 5 — Spearman’s correlation heatmap based on population abundance and environmental variables for families with abundance >1%. AN, available nitrogen; TN, total nitrogen; RAK, rapidly available potassium; RAP, rapidly available phosphorus; TP, total phosphorus, TK, total potassium. Positive correlations (red), negative correlations (blue). *P < 0.05 and **P < 0.01 for the indicated comparisons. [file Image_5.TIF]
