# Supplementary material for: Rhizosphere Bacterial Community Response to Continuous Cropping of Tibetan Barley
Source: Front Microbiol. 2020 Nov 30;11:551444. doi: 10.3389/fmicb.2020.551444 (PMC7734106; doi:10.3389/fmicb.2020.551444)
Supplement: Supplementary Table 1 — The changes of the 20 most abundant OTUs during continuous cropping. [file Table_1.DOCX]

**Table S1**. The changes of the 20 most abundant OTUs during continuous cropping.

| **OTU ID** | **Taxonomic assignment** | **Relative abundance *** | | | | |
| --- | --- | --- | --- | --- | --- | --- |
|  |  | **CC2Y** | **CC3Y** | **CC4Y** | **CC5Y** | **CC6Y** |
| OTU1 | Unclassified *Exiguobacterium* | 0.98% ± 0.05% b | 1.13% ± 0.62% b | 2.20% ± 0.19% a | 1.09% ± 0.15% b | 2.29% ± 0.10% a |
| OTU2 | *Sphingomonas jaspsi* | 2.17% ± 0.09% a | 1.67% ± 0.61% a | 0.30% ± 0.02% b | 0.38% ± 0.04% b | 0.22% ± 0.04% b |
| OTU3 | Unclassified *Sphingomonadaceae* | 0.85% ± 0.04% b | 0.85% ± 0.09% b | 0.66% ± 0.15% b | 1.57% ± 0.40% a | 0.43% ± 0.01% b |
| OTU4 | Unclassified *Sphingomonas* | 0.58% ± 0.03% c | 0.59% ± 0.03% c | 0.53% ± 0.03% c | 0.79% ± 0.03% a | 0.69% ± 0.06% b |
| OTU5 | sugarcane phytoplasma | 0.28% ± 0.04% b | 0.22% ± 0.06% b | 0.59% ± 0.10% b | 1.20% ± 0.35% a | 1.06% ± 0.18% a |
| OTU6 | Unclassified *Sphingomonas* | 0.43% ± 0.01% c | 0.50% ± 0.03% bc | 0.62% ± 0.08% ab | 0.72% ± 0.09% a | 0.48% ± 0.02% c |
| OTU7 | Unclassified *Acinetobacter* | 0.48% ± 0.04% b | 0.41% ± 0.07% b | 0.61% ± 0.06% a | 0.41% ± 0.03% b | 0.61% ± 0.02% a |
| OTU8 | Unclassified *Sphingomonas* | 0.24% ± 0.02% d | 0.35% ± 0.06% c | 0.65% ± 0.02% a | 0.46% ± 0.04% b | 0.52% ± 0.02% b |
| OTU9 | Unclassified *Pseudonocardiaceae* | 0.00% ± 0.00% c | 0.24% ± 0.17% c | 0.65% ± 0.15% b | 0.26% ± 0.24% c | 1.23% ± 0.18% a |
| OTU10 | Unclassified *Sphingopyxis* | 0.35% ± 0.01% bc | 0.32% ± 0.01% c | 0.37% ± 0.01% b | 0.50% ± 0.04% a | 0.37% ± 0.02% b |
| OTU11 | Unclassified *Xanthomonadaceae* | 0.02% ± 0.04% c | 0.13% ± 0.09% c | 0.48% ± 0.02% b | 0.66% ± 0.05% a | 0.79% ± 0.11% a |
| OTU12 | Unclassified *Nocardioides* | 0.24% ± 0.02% b | 0.29% ± 0.08% b | 0.43% ± 0.04% a | 0.43% ± 0.03% a | 0.41% ± 0.01% a |
| OTU13 | Unclassified *Citrobacter* | 0.30% ± 0.01% bc | 0.26% ± 0.03% c | 0.40% ± 0.02% a | 0.34% ± 0.03% ab | 0.39% ± 0.04% a |
| OTU14 | Unclassified *Brevundimonas* | 0.43% ± 0.02% a | 0.42% ± 0.07% a | 0.25% ± 0.04% b | 0.43% ± 0.04% a | 0.27% ± 0.01% b |
| OTU15 | Unclassified *Erythrobacteraceae* | 0.28% ± 0.04% b | 0.27% ± 0.03% b | 0.33% ± 0.04% b | 0.46% ± 0.02% a | 0.32% ± 0.03% b |
| OTU16 | Unclassified *Oxalobacteraceae* | 0.30% ± 0.04% ab | 0.18% ± 0.12% b | 0.34% ± 0.04% a | 0.26% ± 0.06% ab | 0.30% ± 0.01% ab |
| OTU17 | Unclassified *Sphingomonadaceae* | 0.48% ± 0.02% a | 0.44% ± 0.02% a | 0.18% ± 0.02% b | 0.24% ± 0.05% b | 0.17% ± 0.06% b |
| OTU18 | Unclassified *Pseudomonas* | 0.29% ± 0.07% a | 0.37% ± 0.05% a | 0.31% ± 0.02% a | 0.24% ± 0.02% b | 0.28% ± 0.02% a |
| OTU19 | Unclassified *Nitrosospira* | 0.13% ± 0.01% d | 0.29% ± 0.02% bc | 0.31% ± 0.03% b | 0.66% ± 0.07% a | 0.23% ± 0.03% c |
| OTU20 | *Burkholderiales* bacterium X4 | 0.36% ± 0.02% a | 0.28% ± 0.01% b | 0.29% ± 0.01% b | 0.24% ± 0.03% c | 0.26% ± 0.01% bc |

* Relative abundance are represented as average ± SD obtained across quadruple measurements. Average with different superscript letters are significantly different horizontally (*p* < 0.05).
