# Supplementary material for: Rhizosphere Bacterial Community Response to Continuous Cropping of Tibetan Barley
Source: Front Microbiol. 2020 Nov 30;11:551444. doi: 10.3389/fmicb.2020.551444 (PMC7734106; doi:10.3389/fmicb.2020.551444)
Supplement: Supplementary Table 2 — Relative abundance of families against years of continuous cropping of Tibetan barley. [file Table_2.DOCX]

**Table S2**. Relative abundance of families against years of continuous cropping of Tibetan barley.

| **Taxon** | **r^2^** | ***p* value** | **Correlation** |
| --- | --- | --- | --- |
| *Sphingomonadaceae* | 0.38 | 0.004088409 | Negative |
| *Xanthomonadaceae* | 0.35 | 0.006132347 | Positive |
| *Nocardioidaceae* | 0.66 | 1.47E-05 | Positive |
| *Flavobacteriaceae* | 0.33 | 0.007897892 | Positive |
| *Comamonadaceae* | 0.12 | 0.128577048 | Negative |
| *Pseudomonadaceae* | 0.84 | 1.18E-08 | Positive |
| *Hyphomonadaceae* | 0.73 | 1.38E-06 | Negative |
| *Chitinophagaceae* | 0.69 | 5.08E-06 | Negative |
| *Sphingobacteriaceae* | 0.57 | 0.00012196 | Positive |
| *Cytophagaceae* | 0.70 | 4.79E-06 | Positive |

^*^ Only showed the 10 most abundant families. The relative abundances of families versus the years of continuous cropping were linear regressed using *lm*() function in R software.
