# Supplementary material for: Rhizosphere Bacterial Community Response to Continuous Cropping of Tibetan Barley
Source: Front Microbiol. 2020 Nov 30;11:551444. doi: 10.3389/fmicb.2020.551444 (PMC7734106; doi:10.3389/fmicb.2020.551444)
Supplement: Supplementary Table 3 — Dynamics of ecological function during continuous cropping of Tibetan barley. [file Table_3.DOCX]

**Table S3**. Dynamics of ecological function during continuous cropping of Tibetan barley *.

| **Functional groups** | Relative abundance (%) | | | | |
| --- | --- | --- | --- | --- | --- |
|  | **CC2Y** | **CC3Y** | **CC4Y** | **CC5Y** | **CC6Y** |
| chemoheterotrophy | 34.10±0.44 | 35.45±0.17 | 35.31±0.56 | 37.86±0.14 | 35.81±0.61 |
| aerobic chemoheterotrophy | 32.12±0.54 | 33.69±0.15 | 33.86±0.43 | 35.90±0.02 | 34.19±0.62 |
| animal parasites or symbionts | 3.09±0.15 | 2.64±0.12 | 2.97±0.26 | 2.11±0.16 | 2.87±0.04 |
| aromatic compound degradation | 1.84±0.10 | 2.07±0.24 | 3.03±0.08 | 3.05±0.03 | 3.11±0.19 |
| nitrate reduction | 1.52±0.11 | 1.85±0.15 | 2.08±0.16 | 1.74±0.04 | 1.84±0.11 |
| human pathogens all | 1.92±0.10 | 1.63±0.06 | 1.80±0.16 | 1.41±0.10 | 1.77±0.06 |
| fermentation | 1.87±0.21 | 1.52±0.07 | 1.47±0.05 | 1.21±0.06 | 1.82±0.06 |
| ureolysis | 0.97±0.06 | 1.40±0.11 | 1.64±0.03 | 1.26±0.15 | 1.80±0.07 |
| dark hydrogen oxidation | 1.43±0.07 | 1.43±0.09 | 0.82±0.03 | 0.78±0.03 | 1.02±0.27 |
| nitrate respiration | 0.89±0.07 | 1.02±0.06 | 1.01±0.03 | 1.00±0.04 | 1.02±0.07 |
| nitrogen respiration | 0.89±0.07 | 1.02±0.06 | 1.01±0.03 | 1.00±0.04 | 1.02±0.07 |
| phototrophy | 1.74±0.20 | 0.91±0.04 | 0.78±0.11 | 0.47±0.03 | 0.49±0.06 |
| human gut | 0.88±0.08 | 0.70±0.09 | 0.93±0.04 | 0.63±0.04 | 0.94±0.05 |
| mammal gut | 0.88±0.08 | 0.70±0.09 | 0.93±0.04 | 0.63±0.04 | 0.94±0.05 |
| nitrification | 0.72±0.06 | 0.87±0.05 | 0.78±0.05 | 1.04±0.14 | 0.64±0.10 |
| human pathogens pneumonia | 1.17±0.13 | 0.90±0.05 | 0.69±0.08 | 0.47±0.07 | 0.70±0.02 |
| nitrate denitrification | 0.80±0.07 | 0.83±0.09 | 0.60±0.02 | 0.45±0.02 | 0.57±0.10 |
| nitrite denitrification | 0.80±0.07 | 0.83±0.09 | 0.60±0.02 | 0.45±0.02 | 0.57±0.10 |
| nitrous oxide denitrification | 0.80±0.07 | 0.83±0.09 | 0.60±0.02 | 0.45±0.02 | 0.57±0.10 |
| denitrification | 0.80±0.07 | 0.83±0.09 | 0.60±0.02 | 0.45±0.02 | 0.57±0.10 |
| nitrite respiration | 0.80±0.07 | 0.83±0.09 | 0.60±0.02 | 0.45±0.02 | 0.57±0.10 |
| photoautotrophy | 1.17±0.15 | 0.58±0.02 | 0.73±0.13 | 0.34±0.03 | 0.45±0.03 |
| methanol oxidation | 0.48±0.03 | 0.67±0.02 | 0.66±0.02 | 0.61±0.02 | 0.67±0.05 |
| methylotrophy | 0.48±0.03 | 0.67±0.02 | 0.66±0.02 | 0.61±0.02 | 0.67±0.05 |
| cellulolysis | 0.47±0.01 | 0.49±0.09 | 0.56±0.03 | 0.71±0.05 | 0.80±0.07 |
| chitinolysis | 0.76±0.06 | 0.44±0.04 | 0.36±0.10 | 0.83±0.06 | 0.33±0.03 |
| aerobic ammonia oxidation | 0.32±0.03 | 0.44±0.05 | 0.42±0.03 | 0.63±0.06 | 0.34±0.00 |
| cyanobacteria | 0.67±0.08 | 0.40±0.02 | 0.33±0.03 | 0.24±0.01 | 0.32±0.04 |
| oxygenic photoautotrophy | 0.67±0.08 | 0.40±0.02 | 0.33±0.03 | 0.24±0.01 | 0.32±0.04 |
| photoheterotrophy | 0.89±0.14 | 0.50±0.04 | 0.28±0.04 | 0.23±0.03 | 0.17±0.03 |
| aerobic nitrite oxidation | 0.40±0.03 | 0.43±0.02 | 0.36±0.02 | 0.41±0.08 | 0.30±0.10 |
| hydrocarbon degradation | 0.17±0.01 | 0.28±0.02 | 0.40±0.02 | 0.47±0.03 | 0.42±0.06 |
| aromatic hydrocarbon degradation | 0.17±0.01 | 0.25±0.03 | 0.40±0.02 | 0.44±0.03 | 0.42±0.06 |
| aliphatic non methane hydrocarbon degradation | 0.17±0.01 | 0.25±0.03 | 0.40±0.02 | 0.44±0.03 | 0.42±0.06 |
| plant pathogen | 0.46±0.08 | 0.44±0.09 | 0.10±0.02 | 0.02±0.03 | 0.26±0.08 |
| anoxygenic photoautotrophy S oxidizing | 0.51±0.12 | 0.18±0.01 | 0.40±0.12 | 0.10±0.03 | 0.13±0.01 |
| anoxygenic photoautotrophy | 0.51±0.12 | 0.18±0.01 | 0.40±0.12 | 0.10±0.03 | 0.13±0.01 |
| predatory or exoparasitic | 0.31±0.03 | 0.32±0.10 | 0.15±0.03 | 0.13±0.01 | 0.14±0.02 |
| invertebrate parasites | 0.29±0.06 | 0.31±0.03 | 0.24±0.07 | 0.06±0.04 | 0.16±0.01 |
| nitrogen fixation | 0.17±0.05 | 0.19±0.07 | 0.19±0.03 | 0.18±0.06 | 0.26±0.04 |
| ligninolysis | 0.28±0.01 | 0.19±0.00 | 0.16±0.02 | 0.13±0.00 | 0.15±0.02 |
| intracellular parasites | 0.07±0.05 | 0.12±0.10 | 0.08±0.03 | 0.08±0.02 | 0.09±0.00 |
| dark iron oxidation | 0.18±0.03 | 0.08±0.00 | 0.01±0.02 | 0.13±0.01 | 0.01±0.03 |
| dark oxidation of sulfur compounds | 0.00±0.00 | 0.02±0.05 | 0.09±0.02 | 0.02±0.05 | 0.14±0.01 |
| respiration of sulfur compounds | 0.15±0.04 | 0.04±0.01 | 0.05±0.01 | 0.01±0.02 | 0.02±0.03 |
| xylanolysis | 0.00±0.00 | 0.12±0.08 | 0.10±0.04 | 0.01±0.01 | 0.01±0.01 |
| sulfate respiration | 0.12±0.04 | 0.04±0.01 | 0.05±0.01 | 0.01±0.02 | 0.02±0.03 |
| manganese oxidation | 0.04±0.04 | 0.00±0.00 | 0.00±0.00 | 0.00±0.00 | 0.00±0.00 |
| dark thiosulfate oxidation | 0.00±0.00 | 0.00±0.00 | 0.00±0.00 | 0.00±0.00 | 0.01±0.01 |
| sulfur respiration | 0.03±0.02 | 0.00±0.00 | 0.00±0.00 | 0.00±0.00 | 0.00±0.00 |
| thiosulfate respiration | 0.03±0.02 | 0.00±0.00 | 0.00±0.00 | 0.00±0.00 | 0.00±0.00 |

* Values are represented as mean±SDs obtained across quadruplicate measurements.
