# Supplementary material for: Rhizosphere Bacterial Community Response to Continuous Cropping of Tibetan Barley
Source: Front Microbiol. 2020 Nov 30;11:551444. doi: 10.3389/fmicb.2020.551444 (PMC7734106; doi:10.3389/fmicb.2020.551444)
Supplement: Supplementary Table 4 — Relative abundance of ecological function against years of continuous cropping of Tibetan barley. [file Table_4.DOCX]

**Table S4**. Relative abundance of ecological function against years of continuous cropping of Tibetan barley ^*^.

| **Functional groups** | **r^2^** | ***p* value** | **Correlation** |
| --- | --- | --- | --- |
| cellulolysis | 0.84 | <0.0001 | Positive |
| photoheterotrophy | 0.81 | <0.0001 | Negative |
| phototrophy | 0.76 | <0.0001 | Negative |
| aromatic compound degradation | 0.73 | <0.0001 | Positive |
| aromatic hydrocarbon degradation | 0.72 | <0.0001 | Positive |
| aliphatic non methane hydrocarbon degradation | 0.72 | <0.0001 | Positive |
| ureolysis | 0.71 | <0.0001 | Positive |
| ligninolysis | 0.69 | <0.0001 | Negative |
| hydrocarbon degradation | 0.69 | <0.0001 | Positive |
| methanol oxidation | 0.62 | <0.0001 | Positive |
| methylotrophy | 0.62 | <0.0001 | Positive |
| cyanobacteria | 0.59 | <0.0001 | Negative |
| oxygenic photoautotrophy | 0.59 | <0.0001 | Negative |
| human pathogens pneumonia | 0.58 | <0.0001 | Negative |
| predatory or exoparasitic | 0.58 | <0.0001 | Negative |
| nitrate respiration | 0.55 | 0.0002 | Positive |
| nitrogen respiration | 0.55 | 0.0002 | Positive |
| photoautotrophy | 0.55 | 0.0002 | Negative |
| respiration of sulfur compounds | 0.55 | 0.0002 | Negative |
| aerobic chemoheterotrophy | 0.53 | 0.0003 | Positive |
| sulfate respiration | 0.52 | 0.0003 | Negative |
| chemoheterotrophy | 0.51 | 0.0004 | Positive |
| dark oxidation of sulfur compounds | 0.47 | 0.0009 | Positive |
| nitrate denitrification | 0.46 | 0.001 | Negative |
| nitrite denitrification | 0.46 | 0.001 | Negative |
| nitrous oxide denitrification | 0.46 | 0.001 | Negative |
| denitrification | 0.46 | 0.001 | Negative |
| nitrite respiration | 0.46 | 0.001 | Negative |
| invertebrate parasites | 0.41 | 0.0024 | Negative |
| nitrate reduction | 0.41 | 0.0024 | Positive |

^*^ Only showed the functional groups with R^2^ > 0.4 and *P* < 0.05. The relative abundances of functional group versus the years of continuous cropping were linear regressed using *lm*() function in R software.
